# Opsoclonus revealing underlying multiple system atrophy: a case report

**DOI:** 10.1186/s40981-026-00865-5

**Published:** 2026-07-03

**Authors:** Hiromi Kawano, Shotaro Sakimura, Ken Yamaura

**Affiliations:** 1 Department of Anesthesiology, Fukuoka City Hospital, Fukuoka, Japan; 2 Department of Anesthesiology, National Hospital Organization Kyushu Medical Center, Fukuoka, Japan; 3 Operating Rooms, Kyushu University Hospital, 3-1-1, Maidashi, Higashi-ku, Fukuoka, 812-8582 Japan; 4 Department of Anesthesiology and Critical Care Medicine, Kyushu University Hospital, Fukuoka, Japan

**Keywords:** Multiple system atrophy, Opsoclonus, Delayed emergence, General anesthesia, Fentanyl

## Abstract

**Background:**

Delayed emergence from general anesthesia has multiple etiologies; in rare cases, it may unmask an unrecognized neurodegenerative disorder.

**Case presentation:**

A 64-year-old man underwent laparoscopic cholecystectomy under general anesthesia. No neurological abnormalities were identified preoperatively. After surgery, response to verbal stimuli was inadequate; however, other extubation criteria were met and he was extubated. Approximately 45 min later, rapid nystagmus-like eye movements consistent with opsoclonus developed, initially oscillating horizontally and then vertically, and persisted for about six minutes. A similar episode recurred 125 min after extubation. Head computed tomography showed no acute lesions but revealed marked cerebellar and brainstem atrophy. Subsequent neurological evaluation, including magnetic resonance imaging demonstrating the “hot cross bun” sign, fulfilled the Movement Disorder Society diagnostic criteria for clinically established multiple system atrophy with predominant parkinsonism.

**Conclusions:**

In unexplained delayed emergence from general anesthesia, opsoclonus should prompt evaluation for an underlying neurodegenerative disorder.

**Supplementary Information:**

The online version contains supplementary material available at https://doi.org/10.1186/s40981-026-00865-5.

## Background

Delayed emergence from general anesthesia and the development of neurological abnormalities require consideration of a broad range of differential diagnoses. Neurological abnormalities that are not apparent preoperatively can make diagnosis particularly challenging.

We report a case in which marked nystagmus-like eye movements (opsoclonus) were observed in the postoperative period after general anesthesia. Subsequent evaluation led to a postoperative diagnosis of multiple system atrophy (MSA).

## Case presentation

A 64-year-old man (height 164 cm, weight 61 kg) was scheduled to undergo laparoscopic cholecystectomy for symptomatic cholelithiasis. He had a history of choledocholithiasis and septic cholangitis one year earlier. The American Society of Anesthesiologists physical status classification was class II because of mild liver dysfunction (aspartate aminotransferase 79 IU/L and alanine aminotransferase 158 IU/L) and a slightly prolonged prothrombin time (international normalized ratio 1.21). No neurological abnormalities were identified during the preoperative examination.

Anesthesia was induced with fentanyl, propofol, and rocuronium and maintained with desflurane and remifentanil. The surgery lasted 109 min and proceeded without complications.

At the end of surgery, remifentanil and desflurane were discontinued, and sugammadex 120 mg (approximately 2.0 mg/kg) was administered because neuromuscular monitoring showed a train-of-four count of 2. Approximately 30 min after surgery, verbal response and eye opening remained inadequate. However, the cough reflex had recovered, spontaneous ventilation was adequate, oxygenation and ventilation were stable, and hemodynamic status was stable. Neuromuscular recovery was also considered adequate after sugammadex administration. Although incomplete awakening raised concern regarding residual anesthetic effect and aspiration risk, extubation was performed because immediate airway protection and respiratory function were judged to be sufficient under close postoperative monitoring.

Extubation parameters were as follows: blood pressure 130/80 mmHg, heart rate 60 beats per minute, respiratory rate 16 breaths per minute, peripheral oxygen saturation 100%, end-tidal carbon dioxide 30 mmHg, tidal volume 350 mL, end-tidal desflurane 0.7%, and train-of-four ratio 0.9. An anesthetic timeline is provided in Additional file 1: Figure S1.

After extubation, the level of consciousness remained depressed; however, pupillary size and both light and corneal reflexes were normal. Over the subsequent 30 min, consciousness gradually improved, and the patient was able to follow simple commands, such as handgrip and nodding.

**Fig. 1:**
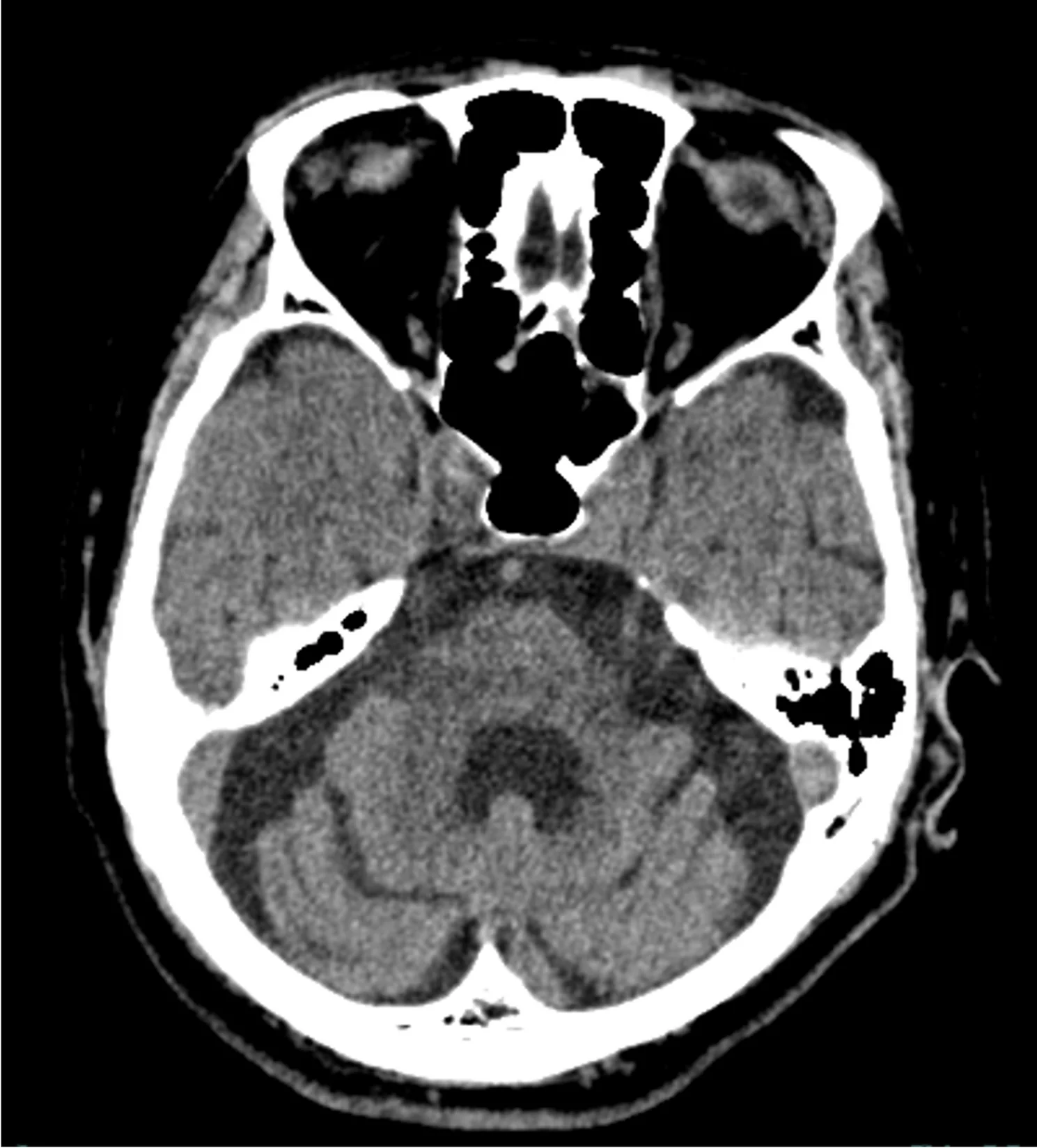
Postoperative head computed tomography Non-contrast head computed tomography obtained postoperatively shows marked cerebellar and brainstem atrophy without evidence of acute intracranial hemorrhage or ischemic lesions

However, 45 min after extubation, rapid, conjugate, multidirectional nystagmus-like eye movements developed. These movements initially occurred horizontally from left to right and were followed by vertical movements from up to down, persisting for approximately six minutes in total. Neurology consultation was obtained. Head computed tomography showed no evidence of intracranial hemorrhage or infarction. Notably, marked atrophy extending from the cerebellum to the brainstem was observed, raising suspicion of underlying spinocerebellar degeneration (Fig. 1).

**Fig. 2:**
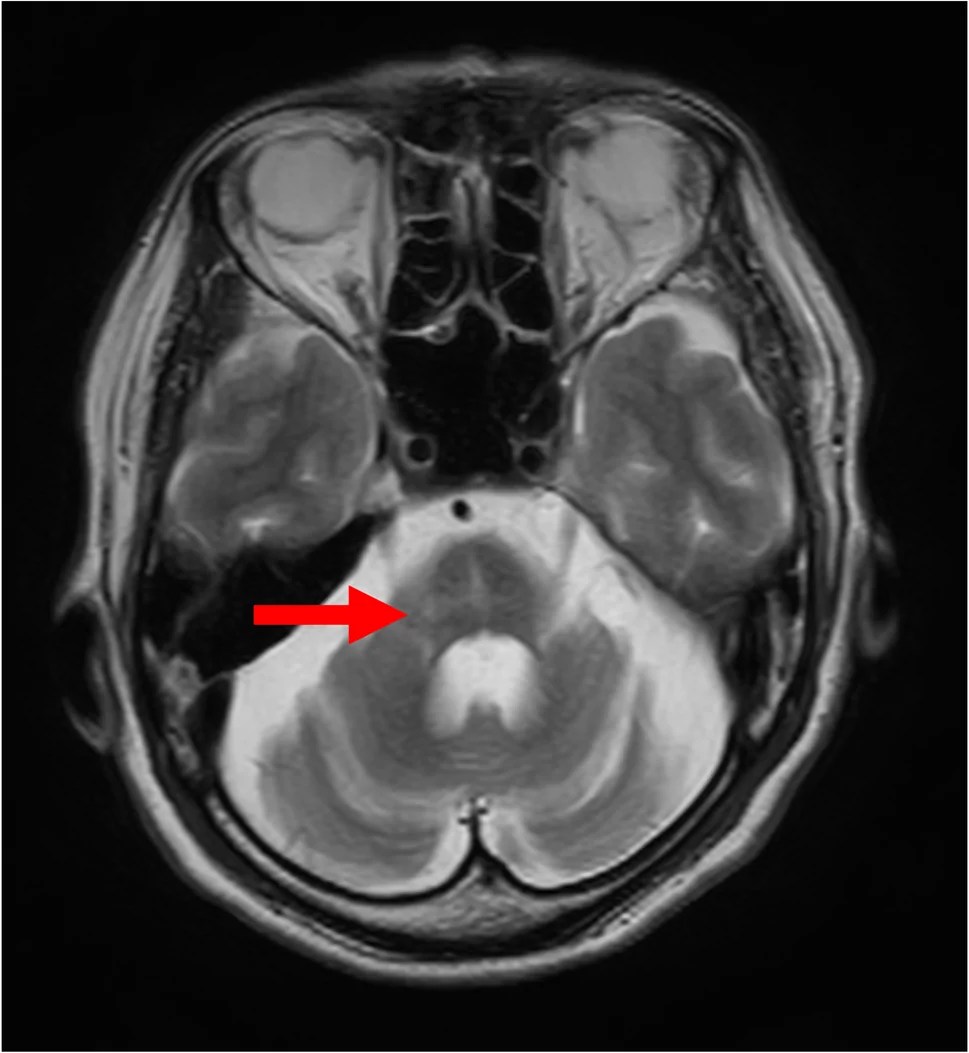
“Hot cross bun” sign on magnetic resonance imaging. Axial T2-weighted brain magnetic resonance imaging demonstrates the characteristic “hot cross bun” sign in the pons (arrow), a supportive neuroimaging feature of multiple system atrophy

He was able to direct his gaze toward nursing staff in response to verbal prompts and could perform handgrip, dorsiflexion, and flexion–extension movements. However, when prompted to speak, he could only produce vocalizations such as “ah” and was unable to state his name. He responded to questions by nodding or shaking his head. During the postoperative examination, similar abnormal eye movements recurred at approximately 125 min after extubation. These movements were clinically diagnosed as opsoclonus (Additional file 2: Video S1; Additional file 3: Video S2) and lasted for about one minute. Even during the episode of opsoclonus, the patient remained able to follow commands, including repetitive hand opening and closing movements. Arterial blood gas analysis obtained at the time of this recurrence showed sodium 140 mmol/L, potassium 4.0 mmol/L, and glucose 124 mg/dL, ruling out a metabolic cause. On postoperative day 3, neurological evaluation during the same hospitalization identified several abnormalities that had not been recognized on preoperative examination, including orthostatic hypotension, masked facies with hypophonia, dysarthria, hypermetric saccades, impaired finger tapping, bilateral limb dysmetria, a positive Romberg sign, and gait ataxia. Brain magnetic resonance imaging performed on the same day revealed the “hot cross bun” sign (Fig. 2). Based on these neurological and imaging findings, the patient met the Movement Disorder Society diagnostic criteria for clinically established MSA with predominant parkinsonism [1]. The patient was discharged on postoperative day 5.

## Discussion

The most striking feature of this case was the postoperative emergence of opsoclonus, which was captured on video (Additional file 2: Video S1; Additional file 3: Video S2) and confirmed by subsequent neurological evaluation. Opsoclonus is characterized primarily by horizontal burst movements, consisting of violent, continuous saccadic eye movements that recur intermittently with marked oscillation. Vertical, torsional, or oblique components may also be present [2]. These movements are often exacerbated by attempted fixation and may persist for several seconds to tens of seconds or longer.

The underlying mechanism is thought to involve dysfunction of inhibitory cerebellar and brainstem control over saccadic eye movements, despite preserved function of the paramedian pontine reticular formation [3]. The etiologies vary by age group. In children, opsoclonus is commonly associated with tumors, including neuroblastoma, and with infections. In adults, it has also been linked to brainstem encephalitis, hypoxemia, cerebrovascular disease, and drug-induced causes, including agents such as diazepam, ketamine, pethidine, and fentanyl [2, 4, 5]. Delayed emergence after general anesthesia is more commonly attributable to residual anesthetic, opioid, or neuromuscular blocking effects, hypoventilation with hypercapnia, hypoxemia, hypoglycemia, electrolyte or other metabolic abnormalities, and acute intracranial events such as stroke or hemorrhage. In the present case, major ventilatory causes were considered less likely because spontaneous ventilation was adequate at extubation, with a respiratory rate of 16 breaths per minute, tidal volume of 350 mL, peripheral oxygen saturation of 100%, and end-tidal carbon dioxide of 30 mmHg. Residual neuromuscular blockade was also considered less likely because the train-of-four ratio had recovered to 0.9 after sugammadex administration. Although a residual volatile anesthetic effect could not be completely excluded, the end-tidal desflurane concentration at extubation was 0.7%. In addition, head computed tomography showed no evidence of acute intracranial hemorrhage or infarction. Metabolic causes such as hypoglycemia and electrolyte abnormalities are also important differential diagnoses in delayed emergence. In this case, however, the subsequent appearance of opsoclonus and the characteristic cerebellar and brainstem findings shifted the diagnostic focus toward an underlying neurodegenerative disorder.

Initially, drug-induced effects were suspected. In the present case, fentanyl may have contributed to the development of opsoclonus. The estimated fentanyl effect-site concentration at the time the nystagmus-like eye movements appeared was approximately 0.48 ng/mL, as calculated using AnestAssist™ pharmacokinetic/pharmacodynamic simulation software based on the Shafer pharmacokinetic model [6] (Additional file 4: Figure S2). However, whether opsoclonus can be induced at this level of fentanyl effect-site concentration remains unclear. The end-tidal desflurane concentration at extubation was 0.7%; however, its relevance to the subsequent abnormal eye movements is also uncertain. Alcohol consumption and hypoxemia are also recognized causes of opsoclonus; however, in this patient, alcohol intake had been discontinued six months before surgery, and hypoxemia was considered unlikely based on the anesthetic course. Another possible explanation is underlying MSA-related cerebellar oculomotor dysfunction. Opsoclonus–myoclonus syndrome associated with MSA has been reported previously, although it is rare [7]. Taken together, these findings suggest that the opsoclonus observed in this case may have been related to cerebellar oculomotor dysfunction associated with MSA and may have been unmasked by general anesthesia.

MSA is a progressive neurodegenerative disorder characterized by the accumulation of insoluble α-synuclein in neurons and oligodendroglia. The disease leads to neuronal loss in the olivopontocerebellar, nigrostriatal, and autonomic nervous systems. According to the Movement Disorder Society diagnostic criteria, MSA is classified on the basis of core clinical features and supportive findings, including characteristic neuroimaging abnormalities [1].

The major clinical manifestations involve five symptom domains: cerebellar features, parkinsonism, autonomic dysfunction, pyramidal signs, and cognitive or psychiatric symptoms [8]. Cerebellar features include oculomotor dysfunction. During the early to middle stages of the disease, cerebellar symptoms, parkinsonism, and autonomic dysfunction predominate; as the disease progresses, these manifestations increasingly overlap. Although MSA had not been diagnosed preoperatively, the patient likely had underlying disease, and the postoperative opsoclonus may have been related to anesthetic agents used during surgery and may have reflected cerebellar oculomotor dysfunction associated with MSA that may have been unmasked by general anesthesia [7, 8].

The presence of sleep apnea warrants particular attention during preoperative evaluation for MSA. Sleep apnea in this condition may result from central alveolar hypoventilation caused by dysfunction of the respiratory centers or anterior horn cells in the medulla and upper thoracic spinal cord, in combination with upper airway obstruction [9, 10].

One major cause of upper airway obstruction is vocal cord abductor paralysis, in which selective neurogenic atrophy and paralysis of the posterior cricoarytenoid muscles impair inspiration despite preserved phonation, leading to high-pitched inspiratory laryngeal stridor, a characteristic feature of MSA [9, 11]. Upper airway obstruction may also occur secondary to vocal cord fixation resulting from increased muscle tone related to supranuclear dysfunction of the intrinsic laryngeal muscles, or from obstruction caused by laryngeal spasm or the epiglottis [9, 10].

Cases have been reported in which patients with MSA developed bilateral vocal cord abductor paralysis after general anesthesia [12], leading to difficult extubation and the need for tracheostomy. For example, Lim and Kennedy described a patient with previously unrecognized MSA who developed complete upper airway obstruction during both induction and emergence from anesthesia and subsequently required ventilatory support and tracheostomy [13]. Postoperative vigilance is also necessary for complications such as carbon dioxide narcosis during oxygen therapy and aspiration related to dysphagia [10].

In summary, we managed a case in which prominent opsoclonus was observed in the postoperative period after general anesthesia and led to the postoperative diagnosis of MSA. When such abnormal eye movements are observed in the perioperative period, neurodegenerative diseases should be considered in the differential diagnosis, in addition to intracranial hemorrhage and cerebral infarction. Furthermore, patients with MSA are at increased risk of upper airway obstruction resulting from vocal cord abductor paralysis. Therefore, thorough preoperative respiratory assessment, careful airway management during extubation, and vigilant postoperative respiratory monitoring are essential [10, 12, 13].

## Supplementary Information

Additional file 1: Figure S1. Timeline of anesthetic management. This file presents a timeline of administered anesthetic agents and key perioperative events. It shows the timing of extubation as well as the onset and recurrence of abnormal eye movements for reference. This material is descriptive and does not imply a causal relationship between anesthetic agents and neurological manifestations. SpO2, peripheral oxygen saturation.

Additional file 2: Video S1. Rapid, conjugate, multidirectional saccadic eye oscillations consistent with opsoclonus observed 45 minutes after extubation.

Additional file 3: Video S2. Recurrence of opsoclonus observed 125 minutes after extubation.

Additional file 4: Figure S2. Simulated fentanyl concentration profile. Estimated fentanyl plasma and effect-site concentrations over time, simulated using AnestAssist™ pharmacokinetic/pharmacodynamic software based on the Shafer pharmacokinetic model, are shown. The estimated effect-site concentration at the onset of abnormal eye movements was approximately 0.48 ng/mL. Values are model-based estimates and do not represent measured plasma concentrations. PK, pharmacokinetic; Pt, patient.

## Data Availability

Data sharing is not applicable to this article as no datasets were generated or analyzed.

## Abbreviations

CT: computed tomography
EtCO2: end-tidal carbon dioxide
MRI: magnetic resonance imaging
MSA: multiple system atrophy
MSA-P: multiple system atrophy with predominant parkinsonism
PK/PD: pharmacokinetic/pharmacodynamics
SpO2: peripheral oxygen saturation
TOF: train-of-four
